# A first comprehensive dataset of the large branchiopods (Branchiopoda, Anostraca, Notostraca, Laevicaudata, Spinicaudata, Cyclestherida) of India

**DOI:** 10.3897/BDJ.14.e182079

**Published:** 2026-04-14

**Authors:** Prashant M Katke, Avinash Isaac Vanjare, Karuthapandi M, Sameer M Padhye

**Affiliations:** 1 Freshwater Invertebrate Biology Laboratory, Department of Zoology, Ahmednagar College, Ahilyanagar-414001, Ahilyanagar, India Freshwater Invertebrate Biology Laboratory, Department of Zoology, Ahmednagar College, Ahilyanagar-414001 Ahilyanagar India; 2 Freshwater Biology Regional Centre, Zoological Survey of India, Hyderabad – 500048, Hyderabad, India Freshwater Biology Regional Centre, Zoological Survey of India, Hyderabad – 500048 Hyderabad India; 3 Biologia Life Science LLP, Savedi, Ahilyanagar -414003, Ahilyanagar, India Biologia Life Science LLP, Savedi, Ahilyanagar -414003 Ahilyanagar India; 4 Centre for Biodiversity Genomics, University of Guelph, Guelph, ON N1G 2W1, Canada, Guelph, Canada Centre for Biodiversity Genomics, University of Guelph, Guelph, ON N1G 2W1, Canada Guelph Canada

**Keywords:** branchiopods, conservation, freshwater, flagship species, Maharashtra, occurrence, temporary waterbodies

## Abstract

**Background:**

Large branchiopod crustaceans play a crucial ecological and economic role as flagship species in temporary aquatic habitats. With less than 10% of the global fauna identified in the Indian subcontinent, it is essential to conduct comprehensive research that includes open access information on their diversity and distribution. Such data generation is vital as it provides key insights to support management and conservation efforts. Given the limited, fragmented and incomplete information on the diversity and distribution of large branchiopods in India, we offer the first comprehensive compilation of species occurrence records for the country. This compilation integrates data from our field collections (2020-2024) and relevant information extracted from literature (from 1859-2024). We provide 581 comprehensive records of 46 species from India, revising the names and taxonomic classification of certain species to highlight the significance of detailed, georeferenced occurrence data in improving our knowledge of large branchiopod biodiversity and distribution. Additionally, we introduce the Rshiny application, which illustrates the spatial distribution of all large branchiopod species found in India, along with their frequency across various habitat types.

**New information:**

We provide a total of 581 open access records using a literature review and our original data from India (1859-2024), with 13 families, 15 genera and 46 species from all five orders (Anostraca, Notostraca, Laevicaudata, Spinicaudata and Cyclestherida). Each record has its current scientific name, location name, geographic information system (GIS) data of the location, date/year of collection and the waterbody type in which the species was found.

Few secondary occurrences date back to more than 150 years and it is likely that these habitats (such as pools and ponds) are now destroyed; the coordinates we provide for many such habitats/regions can help in re-surveying and re-describing certain species or in future research on this group. Following the initial study, which documented 42 species from the Indian subcontinent and 38 from India, we have added four new records to the Indian checklist. A new fairy shrimp species, *Streptocephalus warliae* Katke, Padhye & Vanjare, 2025 was also identified from our samples. An updated RShiny app, entitled 'LbranchidistributR' is presented for visualising the spatial distribution of all large branchiopod species occurring in India, along with the frequency of occurrence in different habitat types.

## Introduction

The Indian subcontinent is a vast and biogeographically diverse region harbouring many unique organisms (Venkataraman and Sivaperuman 2018). However, research efforts have consistently focused on 'attractive' and larger fauna (mammals, birds, reptiles and amphibians) rather than invertebrates, including freshwater branchiopod crustaceans.

Freshwater habitats harbour rich faunal biodiversity worldwide (Dudgeon et al. 2006). Large and permanent aquatic ecosystems, owing to their visibility and ease of access, have been more comprehensively studied than temporary water habitats (e.g. rock pools, ditches, vernal pools and ponds), likely because they have often been ignored as barren and unproductive (Watve 2013, Dixneuf et al. 2021). Nevertheless, a high diversity of invertebrate fauna exists there (Williams 1997, Colburn et al. 2008), despite the challenges posed by extreme temperatures, erratic rainfall, limited food supply, high predation rates and competition (Bozelli et al. 2008).

Invertebrates, such as large branchiopods, are ecologically and economically significant, playing vital roles in aquatic food chains, aquaculture, water quality assessment, biomonitoring and biological control (Kulkarni et al. 2015, Rogers et al. 2020, Rogers 2024). Large branchiopods, an ancient group of crustacean invertebrates known for their role as flagship species in temporary habitats, have not been extensively studied in India (Rogers and Padhye 2015). Although research on large branchiopods began in the 1850s, of the approximately 500 recognised species (Rogers and Padhye 2015), only 42 and 38 have been documented in the Indian subcontinent and India, respectively (Vanjare et al. 2024).

Information on their diversity and occurrence in India is limited and incomplete; however, most species have a restricted distribution and show cryptic speciation (Rogers and Padhye 2015). However, the study did not comment on the detailed occurrence, georeference data and distribution of the species and a few new species and records have been added to the list after 2015 (Padhye and Ghate 2016, Padhye et al. 2018, Padhye et al. 2023, Katke et al. 2025).

Adverse anthropogenic activities, especially in developing nations such as India, have caused an unprecedented rapid decline in freshwater ecosystems (Lynch et al. 2023). Invertebrate diversity, especially that of large branchiopods, may be threatened by climate change (Waterkeyn et al. 2009). Baseline surveys, biodiversity assessments and secondary source data are crucial because they provide essential information to assist in management and conservation strategies and guide decision-making (Weaver et al. 2008, Marques et al. 2024, Telletxea et al. 2025). Baseline data will be invaluable to future researchers and will shape conservation efforts.

Thus, this study aimed at providing detailed information on 46 large branchiopod species in India, based on published scientific sources and our own collections.

## General description

### Purpose

The present study attempts to provide information on large branchiopods from India. This study includes both original data (author collections) and secondary data (from research papers). An RShiny application called 'LbranchidistributR' is also presented. Such baseline information on diversity, secondary source data, occurrence, and georeferencing could help support future biodiversity research and conservation (Giannini et al. 2025).

## Sampling methods

### Sampling description

**Figure 1.**
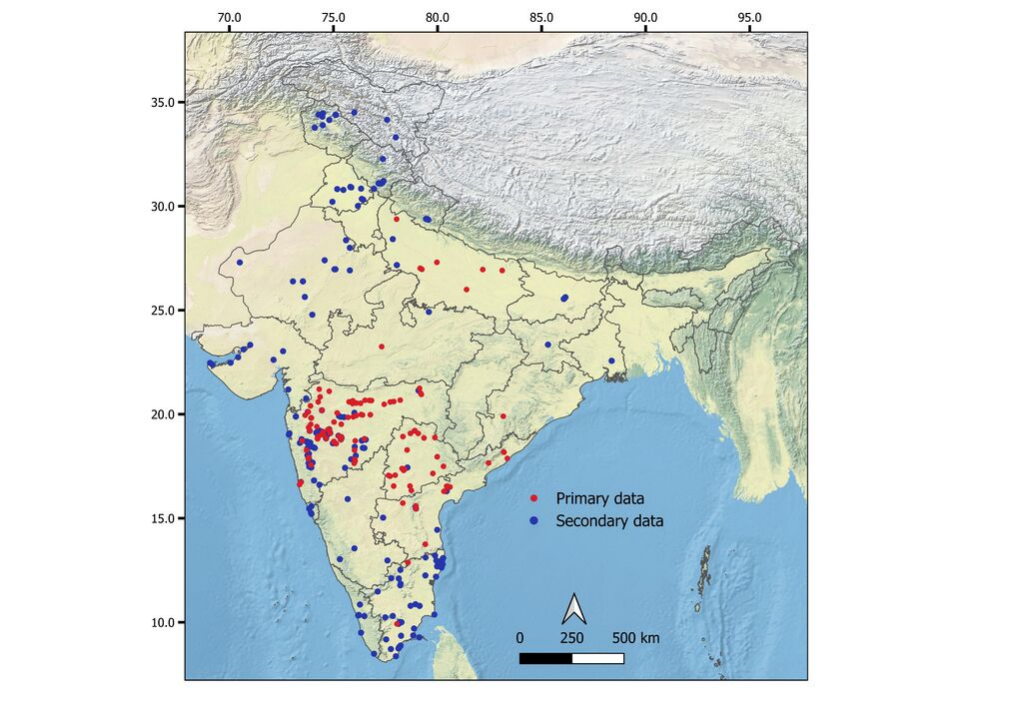
Map indicating original collection (red circle) and secondary data (blue circle) of large branchiopod occurrences in India.

Data were collected using primary and secondary methods (Suppl. material 1). Primary data included actual sampling records from our collections (Fig. 1, Suppl. material 1). Sampling was carried out at each site using a 150-micron hand net, followed by preservation in absolute alcohol. Specimens were identified to the genus and/or species level under a stereomicroscope and further under a compound microscope using standard literature (detailed description in Vanjare et al. (2024)), consultation with experts and available keys (see Rogers and Olesen (2016), Padhye et al. (2018), Sanoamuang et al. (2020), Padhye et al. (2023), Vanjare et al. (2024), Katke et al. (2025)). Species names are given according to recent taxonomic literature (see Vanjare et al. (2024)). The specimens were stored at Zoological Survey of India (ZSI), Pune, Ahmednagar College Ahilyanagar and ZSI, Hyderabad.

**Figure 2.**
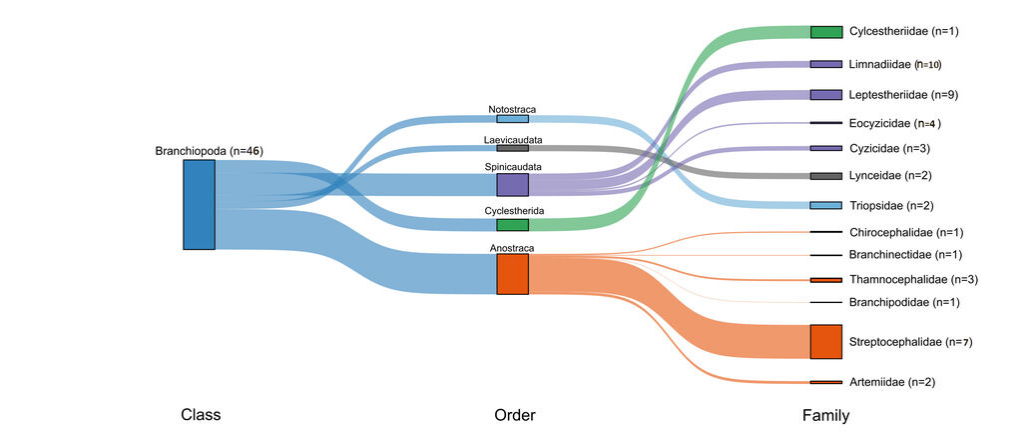
Sankey plot indicating the class, order and families and number of species of large branchiopods from the original collection and secondary data.

Secondary data involved a thorough literature search using public databases (Scopus, Google Scholar, the Biodiversity Heritage Library, ResearchGate and Web of Science). Species records from 1859-2024 were included as secondary data. Location names were retrieved from the original references and their GIS data were obtained using Google Earth (https://earth.google.com/web/). The resolution of the GIS data was based on the locality information (Ex. Locality name as Nagpur, a city rather than exact site-based information). A map of the study region (Fig. 1) was prepared using QGIS software. A Sankey diagram was constructed using the 'ggsankey' package in R using the class, order and family level data and respective species numbers in each category (Fig. 2). Overall, 581 entries (Suppl. material 1) were generated during the study period (1859-2024). Names and taxonomic position of certain species were updated (see Rogers and Padhye (2015), Rogers and Olesen (2016), Rogers (2020), Ghodke et al. (2023), Vanjare et al. (2024), Rogers (2024).

A Rshiny (Chang W et al. 2025) app, 'LbranchidistributR' built for visualising large branchiopod distribution on the Indian subcontinent (https://github.com/sameerpadhye/Large_branchiopoda_distribution.git) was modified and updated based on the new records (Fig. 3). The app has a simple graphical user interface for visualising the spatial distribution of all species of large branchiopods occurring on the Indian subcontinent via a leaflet map (Cheng et al. 2025) along with the frequency of occurrence in different habitat types (pools, ponds, lakes/reservoirs, salt lakes, rivers). The app is hosted on https://sameermpadhye.shinyapps.io/LbranchidistributR/ and the code is open source and available on Github https://github.com/sameerpadhye/Large_branchiopoda_distribution.git.

## Geographic coverage

### Description

The original collection data are mostly from the semi-arid central and western parts of India, with a few records from the eastern and northern regions. Secondary data cover most parts of India. Few states, especially those in the north-eastern region, have no or limited sampling.

### Coordinates

8°4' and 37°6' Latitude; 68°7' and 97°25' Longitude.

## Taxonomic coverage

### Description

Rogers and Padhye (2015) reported 38 species from India; however, further studies (Padhye and Ghate 2016, Padhye and Rabet 2017, Padhye et al. 2018, Sanoamuang et al. 2020, Padhye et al. 2023, Katke et al. 2025) have taken the tally to 46 species (Table 1, Fig. 2). The orders Anostraca and Spinicaudata were the most diverse, with 15 and 26 species, respectively. The species-rich families included Limnadiidae (n = 10) and Leptestheriidae (n = 8), followed by Streptocephalidae (n = 7). The widespread *Streptocephalus dichotomus* Baird, 1860 has been documented in 139 locations, whereas *Cyclestheria hislopi* (Baird, 1859) has 72 records all over India.

### Taxa included

**Table:** 

Rank	Scientific Name	Common Name
kingdom	Animalia Linnaeus, 1758	Animals
phylum	Arthropoda Gravenhorst, 1843	
class	Branchiopoda Latreille, 1817	
order	Notostraca Sars, 1867	
family	Triopsidae Keilhack, 1909	Tadpole shrimps
order	Anostraca Sars, 1867	
family	Artemiidae Grochowski, 1896	Fairy shrimps
family	Branchinectidae Daday, 1910	Fairy shrimps
family	Streptocephalidae Daday, 1910	Fairy shrimps
family	Chirocephalidae Daday, 1910	Fairy shrimps
family	Thamnocephalidae Packard, 1877	Fairy shrimps
family	Branchipodidae Baird, 1852	Fairy shrimps
order	Laevicaudata Linder, 1945	
family	Lynceidae Stebbing, 1902	Smooth clam shrimps
order	Spinicaudata Linder, 1945	
family	Cyzicidae Stebbing, 1910	Clam shrimps
family	Eocyzicidae Schwentner, Rabet, Richter, Giribet, Padhye, Cart, Bonillo & Rogers, 2020	Clam shrimps
family	Leptestheriidae Stebbing, 1902	Clam shrimps
family	Limnadiidae Burmeister, 1843	Clam shrimps
order	Cyclestherida Sars, 1877	
family	Cyclestheriidae Sars, 1877	Tropical clam shrimps

## Usage licence

### Usage licence

Creative Commons Public Domain Waiver (CC-Zero)

## Data resources

### Data package title

Dataset of the large branchiopods (Branchiopoda: Anostraca, Notostraca, Laevicaudata, Spinicaudata, Cyclestherida) of India

### Resource link

https://zenodo.org/records/19335685

### Number of data sets

1

### Data set 1.

#### Data set name

Katke, P., Vanjare, A., Karuthapandi, M., & Padhye, S. (Authors) (2026). Dataset of the large branchiopods (Branchiopoda: Anostraca, Notostraca, Laevicaudata, Spinicaudata, Cyclestherida) of India [Dataset]. Zenodo. https://doi.org/10.5281/zenodo.19335685

#### Data format

csv

#### Download URL

https://doi.org/10.5281/zenodo.19335685

#### Data format version

Version 5

#### Description

We present 581 original and secondary records of 46 species from India (Anostraca, Notostraca, Laevicaudata, Spinicaudata and Cyclestherida). Every record has a scientific name, location name, coordinate data, year of collection, waterbody type and other relevant information. Data from our own collections, as well as from the studied secondary records are presented here.

**Data set 1.** 

Column label	Column description
occurrenceID	A unique code for each occurrence.
scientificName	Correct scientific name of the species.
institutionCode	Place where preserved specimens are hosted.
basisOfRecord	Primary (original collection) or secondary data (research papers).
kingdom	Kingdom name.
phylum	Phylum name.
class	Class name.
order	Order name.
family	Family name.
genus	Genus name.
specificEpithet	Species name.
taxonRank	Level of identification of the specime (genus/species).
verbatimIdentification	Taxonomic identification as it appeared in the original record.
identificationQualifier	Doubtful species.
eventDate	Year of collection or paper published.
eventRemarks	Information about missing date/year.
locality	Name of the locality, place, city, district from where the specimen was collected.
stateProvince	State or union territory in India.
countryCode	Country where the study was conducted.
habitat	Type of waterbody.
decimalLatitude	The geographic latitude (in decimal degrees) of the geographic centre of the sampling location.
decimalLongitude	The geographic longitude (in decimal degrees) of the geographic centre of the sampling location.
geodeticDatum	Geographic coordinates basis.
recordedBy	Name(s) of the person who collected the specimen.
identifiedBy	Name(s) of the person who identified the specimen.
associatedReferences	List of associated references for each secondary data entry.

## Supplementary Material

09BD46BF-4732-56DC-B9A0-71FC6F30CEFA 10.3897/BDJ.14.e182079.suppl1 Supplementary material 1 Occurrence data of large branchiopods of India Data type Occurrence data File: oo_1567280.txt https://binary.pensoft.net/file/1567280 Prashant M. Katke, Avinash Isaac Vanjare, Karuthapandi Madasamy, Sameer M. Padhye

## Figures and Tables

**Figure 3a.**
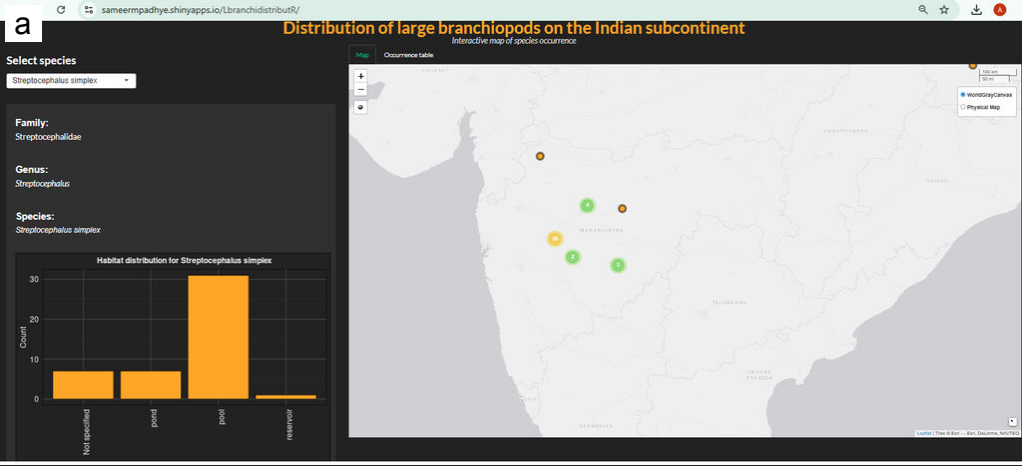
Rshiny app, 'LbranchidistributR' showing distribution of a single species;

**Figure 3b.**
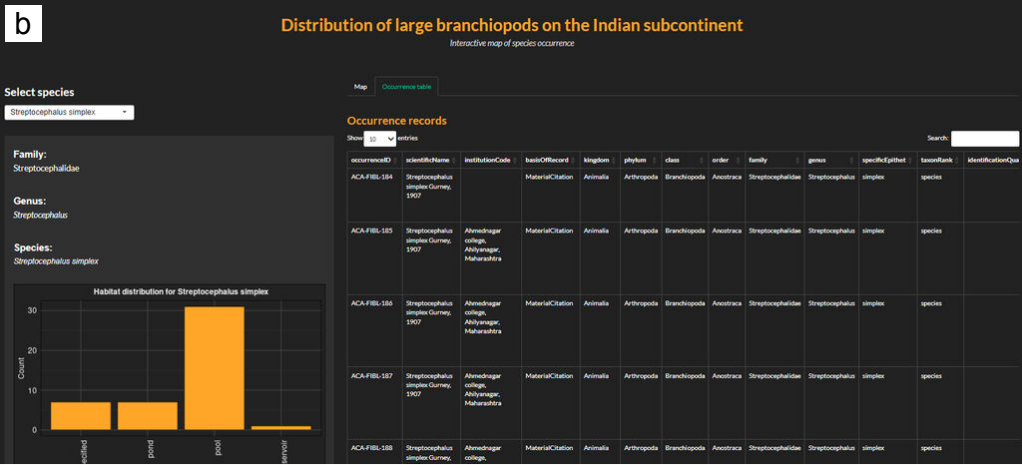
Rshiny app, 'LbranchidistributR' showing details of a single species.

**Table 1.** List of species documented from India, as available from Original (our collections) and other Indian secondary records (available literature).

**Sr. No**	**order**	**family**	**genus**	**species**
1	Anostraca	Artemiidae	Artemia	*Artemia franciscana* Kellogg, 1906
2	Anostraca	Artemiidae	Artemia	*Artemia salina* (Linneaus, 1758)
3	Anostraca	Streptocephalidae	Streptocephalus	*Streptocephalus dichotomus* Baird, 1860
4	Anostraca	Streptocephalidae	Streptocephalus	*Streptocephalus echinus* Bond, 1934
5	Anostraca	Streptocephalidae	Streptocephalus	*Streptocephalus longimanus* Bond, 1934
6	Anostraca	Streptocephalidae	Streptocephalus	*Streptocephalus sahyadriensis* Rogers and Padhye, 2014
7	Anostraca	Streptocephalidae	Streptocephalus	*Streptocephalus simplex* Gurney, 1907
8	Anostraca	Streptocephalidae	Streptocephalus	*Streptocephalus spinifer* Gurney, 1907
9	Anostraca	Streptocephalidae	Streptocephalus	*Streptocephalus warliae* Katke, Padhye & Vanjare, 2025
10	Anostraca	Branchipodidae	Branchipodopsis	*Branchipodopsis affinis* Sars, 1901
11	Anostraca	Thamnocephalidae	Branchinella	*Branchinella hardingi* (Qadri and Baqai, 1956)
12	Anostraca	Thamnocephalidae	Branchinella	*Branchinella maduraiensis* (Raj, 1951)
13	Anostraca	Thamnocephalidae	Carinophallus	*Carinophallus ornata* (Daday, 1910)
14	Anostraca	Branchinectidae	Branchinecta	*Branchinecta orientalis* Sars, 1901
15	Anostraca	Chirocephalidae	Chirocephalus	*Chirocephalus priscus* (Daday, 1910)
16	Notostraca	Triopsidae	Triops	*Triops cancriformis* (Bosc, 1801)
17	Notostraca	Triopsidae	Triops	Triops cf. granarius Lucas, 1864
18	Laevicaudata	Lynceidae	Lynceus	*Lynceus denticulatus* Gurney, 1930
19	Laevicaudata	Lynceidae	Lynceus	*Lynceus indicus* Daday, 1913
20	Spinicaudata	Cyzicidae	Ozestheria	*Ozestheria annandalei* (Daday, 1913)
21	Spinicaudata	Cyzicidae	Ozestheria	*Ozestheria indicus* (Gurney, 1906)
22	Spinicaudata	Cyzicidae	Ozestheria	*Ozestheria ludhianatus* (Battish, 1981)
23	Spinicaudata	Eocyzicidae	Eocyzicus	*Eocyzicus bouvieri* Daday, 1913
24	Spinicaudata	Eocyzicidae	Eocyzicus	*Eocyzicus dhilloni* Battish, 1981
25	Spinicaudata	Eocyzicidae	Eocyzicus	*Eocyzicus hutchinsoni* Bond, 1934
26	Spinicaudata	Eocyzicidae	Eocyzicus	*Eocyzicus plumosus* Royan and Sumitra, 1973
27	Spinicaudata	Leptestheriidae	Leptestheria	*Leptestheria chalukyae* Padhye, Kulkarni, Pagni & Rabet 2023
28	Spinicaudata	Leptestheriidae	Leptestheria	*Leptestheria dumonti* Subash Babu and Bijoy Nandan, 2010
29	Spinicaudata	Leptestheriidae	Leptestheria	*Leptestheria gomantaki* Padhye, Kulkarni, Pagni & Rabet 2023
30	Spinicaudata	Leptestheriidae	Leptestheria	*Leptestheria gurneyi* Padhye and Ghate, 2016
31	Spinicaudata	Leptestheriidae	Leptestheria	*Leptestheria jaisalmerensis* Tiwari, 1962
32	Spinicaudata	Leptestheriidae	Leptestheria	*Leptestheria nobilis* Sars, 1900
33	Spinicaudata	Leptestheriidae	Leptestheria	*Leptestheria sarsi* (Daday, 1923)
34	Spinicaudata	Leptestheriidae	Leptestheria	*Leptestheria simhadrii* (Simhachalam and Timms, 2012)
35	Spinicaudata	Leptestheriidae	Sewellestheria	*Sewellestheria sambharensis* Tiwari, 1966
36	Spinicaudata	Limnadiidae	Eulimnadia	*Eulimnadia azisi* Subash Babu and Bijoy Nandan, 2010
37	Spinicaudata	Limnadiidae	Eulimnadia	*Eulimnadia chaperi* (Simon, 1886)
38	Spinicaudata	Limnadiidae	Eulimnadia	*Eulimnadia compressa* (Baird, 1849)
39	Spinicaudata	Limnadiidae	Eulimnadia	*Eulimnadia cryptus* Sanoamuang, Padhye and Rogers, 2020
40	Spinicaudata	Limnadiidae	Eulimnadia	*Eulimnadia gibba* Sars, 1900
41	Spinicaudata	Limnadiidae	Eulimnadia	*Eulimnadia gunturensis* Radhakrishna and Durga Prasad, 1976
42	Spinicaudata	Limnadiidae	Eulimnadia	*Eulimnadia indocylindrova* Durga Prasad and Simhachalam, 2004
43	Spinicaudata	Limnadiidae	Eulimnadia	*Eulimnadia micheali* Nayar and Nair, 1968
44	Spinicaudata	Limnadiidae	Eulimnadia	*Eulimnadia ovata* Nayar, 1965
45	Spinicaudata	Limnadiidae	Eulimnadia	*Eulimnadia bondi* Padhye, Rabet, Kulkarni & Pagni 2018
46	Cyclestherida	Cyclestheriidae	Cyclestheria	*Cyclestheria hislopi* (Baird,1859)
